# Scale effect of slip boundary condition at solid–liquid interface

**DOI:** 10.1038/srep43125

**Published:** 2017-03-03

**Authors:** Gyoko Nagayama, Takenori Matsumoto, Kohei Fukushima, Takaharu Tsuruta

**Affiliations:** 1 Department of Mechanical Engineering, Kyushu Institute of Technology, Tobata, Kitakyushu, Fukuoka 804-8550, Japan

## Abstract

Rapid advances in microelectromechanical systems have stimulated the development of compact devices, which require effective cooling technologies (e.g., microchannel cooling). However, the inconsistencies between experimental and classical theoretical predictions for the liquid flow in microchannel remain unclarified. Given the larger surface/volume ratio of microchannel, the surface effects increase as channel scale decreases. Here we show the scale effect of the boundary condition at the solid–liquid interface on single-phase convective heat transfer characteristics in microchannels. We demonstrate that the deviation from classical theory with a reduction in hydraulic diameters is due to the breakdown of the continuum solid–liquid boundary condition. The forced convective heat transfer characteristics of single-phase laminar flow in a parallel-plate microchannel are investigated. Using the theoretical Poiseuille and Nusselt numbers derived under the slip boundary condition at the solid–liquid interface, we estimate the slip length and thermal slip length at the interface.

Heat and mass transfer in microchannels has been extensively investigated over the past several decades. Continuum theory is applicable to single-phase liquid flow in microchannels, which implies that the equations developed for macroscale applications, such as the Navier–Stokes equation and convective heat transport equations, will be applicable to even small channels^1^,^2^,^3^,^4^,^5^. In a fully developed laminar channel flow, the classical theoretical solutions for the Nusselt number (hereafter, N_u_; 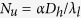, where α is the convective heat transfer coefficient, *D*_*h*_ is the hydraulic diameter of a channel, and λ is the thermal conductivity) and Poiseuille number (hereafter, P_o_; *P*_*o*_ = *fR*_*e*_, where *f* is the Fanning friction factor and R_e_ is the Reynolds number) are constants and are independent of the Reynolds number. Because *αD*_*h*_ is nearly a constant, the forced convection heat transfer coefficients in fully developed laminar channel flows are expected to increase with a decrease in the microchannel size.

Table 1 lists the typical N_u_ and P_o_ values for a fully developed laminar flow in various channels^6^, where N_uT_ is the N_u_ at a uniform surface temperature, and N_uq_ is the N_u_ at a uniform heat flux. The ratio of N_uq_ to N_uT_ is larger than 1. The N_uT_ listed in the table is the lower limit of N_u_ because the axial conduction effect is absent when the surface temperature is uniform. In other words, N_u_ cannot be less than N_uT_ in a thermally developing flow or in a flow under uniform surface heat flux accompanied by axial conduction. However, the experimental N_u_ for the forced convection of a single-phase liquid flow in Si microchannels differs significantly from that predicted using continuum theory^7^,^8^,^9^,^10^,^11^,^12^,^13^,^14^,^15^,^16^,^17^,^18^,^19^,^20^. These significant differences have been mainly attributed to experimental errors pertaining to the effects of axial conduction^16^,^17^,^18^ and roughness^19^,^20^. However, because most studies report similar behaviours (i.e., N_u_ in microchannels decreases with decreasing R_e_), the discrepancies between the theoretical and experimental results cannot be completely attributed to experimental errors. Davis and Gill^21^, who were among the first to examine the axial conduction effect in laminar flow between parallel plates, concluded that the axial conduction effect reduced N_u_. Other researchers^12^,^16^,^17^,^18^ reported that the difference between experimental and theoretical N_u_ increased with decreasing R_e_ in microchannels could be attributed to the effects of axial heat conduction. On the other hand, the analytical results reported by Maranzana *et al*.^17^ and Lin *et al*.^18^ for the effects of axial heat conduction on single-phase microchannel flows yielded a much lower value of N_u_ than the theoretical results, which is in contrast to the conventional predictions of N_uq_ > N_uT_ as the axial conduction increases. Thus, whether N_u_ can be less than N_uT_, which is the lower limit of N_u_ without the axial conduction effect under uniform surface temperature, remains unresolved.

Here we focus on the scale effect on single-phase convective heat transfer in microchannels. We demonstrate that the deviation of N_u_ from that in classical theory with a reduction in the hydraulic diameters of the microchannels is due to solid–liquid interfacial resistance, which can be expressed in terms of the slip length and the thermal slip length (i.e., the Kapitza length). In addition, the convective heat transfer characteristics of single-phase laminar flow in parallel-plate microchannels are investigated experimentally. Finally, using the theoretical P_o_ and N_u_ numbers derived under the slip boundary condition at the solid–liquid interface, we estimate the slip length and thermal slip length at the interface.

## Models and Methods

### Slip boundary condition at solid–liquid interface

**Figure 1:**
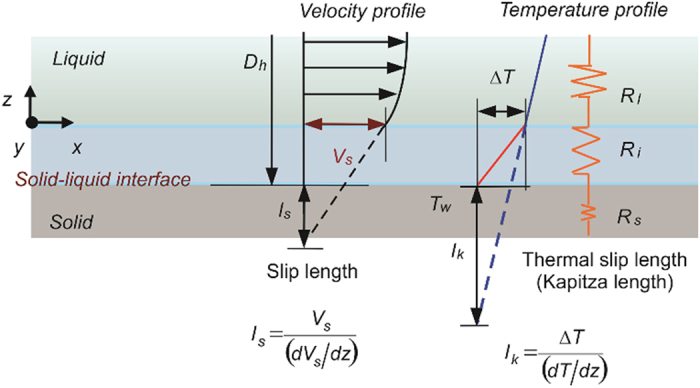
Slip boundary condition at the solid–liquid interface: slip length and thermal slip length.

The boundary condition at the solid–liquid interface is a factor that strongly influences the thermohydraulic characteristics of single-phase liquid flow in microchannels. The continuum boundary condition (i.e., no-slip boundary condition) may fail because of the molecular interactions at the solid–liquid interface, and the slip boundary condition may be significant in nanochannel flow^22^,^23^,^24^,^25^,^26^. According to the Navier’s model, the slip velocity at solid–liquid boundaries is linearly proportional to the velocity gradient at the surface:


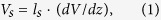


where *l*_*s*_ is the hydrodynamic slip length. Slip length *l*_*s*_ can be obtained by extrapolating the velocity profile from the position at the solid–liquid interface in the fluid to the position at which the velocity becomes zero, as shown in Fig. 1. Analogously, the slip thermal boundary condition can be determined using the “thermal slip length”, that is, the position at which the temperature difference between the liquid and solid is zero. The physical meaning of thermal slip length, also known as the Kapitza length *l*_*k*_, is the thickness of the thermal resistance at the solid–liquid interface:


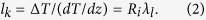


Here, *ΔT* is the temperature jump of the first layer of liquid located at the interface, *dT*/*dz* is the temperature gradient of the liquid, *R*_*i*_ is the thermal resistance at the solid–liquid interface, and *λ*_*l*_ is the thermal conductivity of the liquid.

### Forced convection for fully developed laminar flow under slip boundary condition

**Figure 2:**
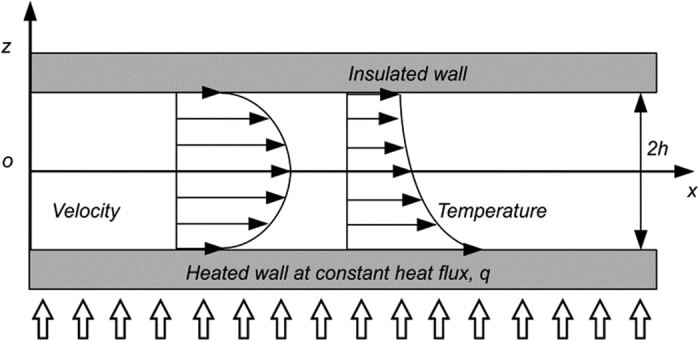
Parallel-plate Poiseuille flow under slip boundary condition.

Consider a parallel-plate Poiseuille flow subjected to constant heat flux at one channel wall in the steady state (Fig. 2). If the spacing between the parallel plates 2*h* is small relative to the size of the parallel plates, the hydraulic diameter is *D*_*h*_ = 4*h*. Assuming that the flow is incompressible and that all of the thermophysical properties are constant, the velocity profile of a hydrodynamic fully developed laminar flow under the slip boundary condition at both channel walls can be derived as follows:


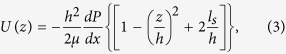


where *μ* is the viscosity and *dP/dx* is the pressure drop. For a fully developed laminar flow, P_o_ is


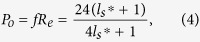


where *l*_*s*_^*∗*^ = *l*_*s*_/(*2h*) and *f* is the fanning friction factor. For *l*_*s*_ = 0 or *l*_*s*_ ≫ *2h*, Eqs (3) and (4) agree with the theoretical predictions under the continuum assumption and P_o_ is a constant (=24). N_u_ can be obtained as


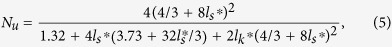


where *l*_*k*_^*∗*^ = *l*_*k*_/(*2h*). For *l*_*s*_ = 0 or *l*_*s*_* *≫* 2h* and *l*_*k*_ = 0 or *l*_*k*_* *≫* 2h*, Eq. (5) agrees with the theoretical prediction under the continuum assumption, and N_u_ is a constant (=5.38).

When the critical dimension of the flow decreases to a size comparable with that of the liquid molecule, *l*_*s*_ and *l*_*k*_ can no longer be ignored, and the slip boundary condition begins to strongly influence the momentum transfer and heat transfer characteristics in the microchannels. In particular, the solid–liquid interfacial resistance is dependent on the molecular interaction, that is, the contact condition between the liquid and the channel wall. Therefore, for a macroscopic smooth wall or a nanostructured wall, the scale effect of the interfacial resistance due to surface roughness and surface wettability becomes increasingly apparent.

## Experiment

### Si-based microchannel test section

**Figure 3:**
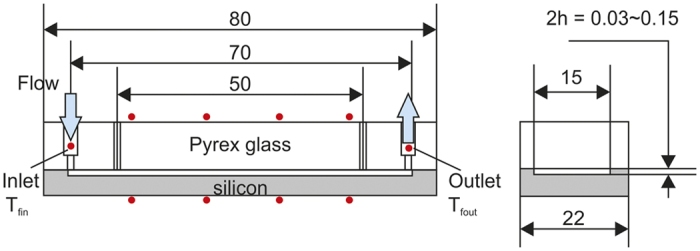
Si microchannel test section.

Si-based microchannels (70 mm (length) × 15 mm (width); 4 channel depths: 30, 50, 100, 150 μm) were prepared through KOH wet-etching of p-type Si wafers in the <100> orientation. The etched microchannels had a rectangular cross-section, as shown in Fig. 3. A Pyrex glass cover was anodically bonded to the Si wafer substrate at 350 °C and 2.0 kV to seal the microchannel, after which the parallel-plate microchannel test section was fabricated. Given that the depths of the microchannels were small relative to their widths and lengths, the channel hydraulic diameter *D*_*h*_ was nearly twice the channel depth (i.e., *D*_*h*_ = 60, 100, 200, and 300 for the four aforementioned channel depths).

To measure the pressure drop, two holes spaced 50 mm apart were fabricated in the cover glass in order to connect to a differential pressure sensor. On the backside of the microchannel, an aluminium thin film heater was sputtered, rendering the Si-based microchannel surface a heated wall subject to constant heat flux. The initial water contact angle at the fresh and clean Si surface was 58° ± 3°, but it decreased to 36° ± 3° because of the oxidation of the thin SiO_2_ film. This surface served as the microchannel surface which the slip boundary condition was applied. The test section was finally assembled, and the bottom surface of the microchannel substrate and the top cover glass surface were well insulated to reduce heat loss from the test section.

### Experimental apparatus

**Figure 4:**
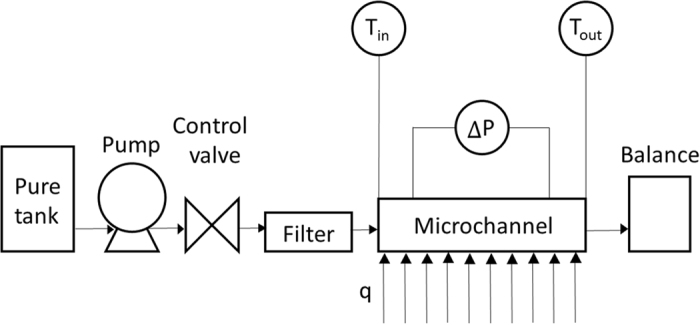
Schematic of experimental apparatus.

The experimental apparatus is shown in Fig. 4, which is consisted of a tank, a pump, valves, the test section of the Si-based microchannel, and a balance. Pure water (Kishida Chemical; electrical resistivity = 18 MΩcm) was used as the working fluid. The fluid temperatures were measured using a T-type thermocouple (diameter = 0.2 mm) at both the inlet and outlet. The wall temperatures were measured using eight T-type thermocouples. All of the pressure and temperature data were collected at 25 °C and 40 RH% by using a data logger and then transmitted to a computer.

## Results and Discussions

### Experimental Poiseuille Number

The friction factor *f* is obtained from the pressure drop 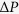, the distance over which the pressure is measured *L* in the fully developed flow region, the fluid density *ρ*, and the mean velocity of the working fluid *U*, as shown in Eq. (6).


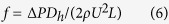


Then, the Po number can be obtained as follows.


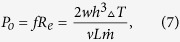


where the microchannel width *w* is 15 mm, 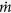 is the mass flow rate, and 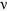 is the dynamic viscosity of the working fluid.

**Figure 5:**
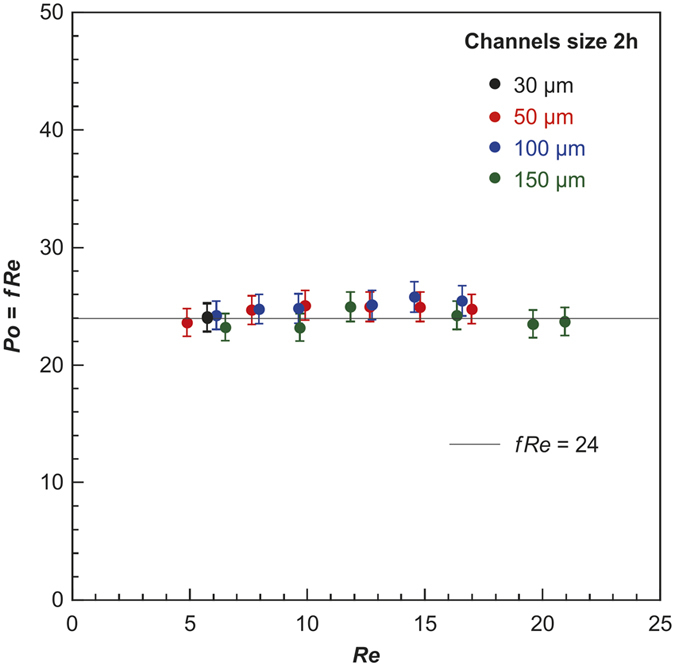
Experimental Poiseuille numbers P_o_ vs. Reynolds number R_e_ in microchannels.

Figure 5 shows the experimental results for the Poiseuille number in the microchannels with hydraulic diameters *D*_*h*_ of 60 μm, 100 μm, 200 μm, and 300 μm, respectively. The results obtained for the Poiseuille number (from more than 3 different independent experiments) agree well with the theoretical values, based on the continuum boundary condition. This could be explained by the surface being covered in a thin, hydrophilic SiO_2_ film and the slip velocity being negligible in the studied cases.

### Experimental Nusselt number

The heat flux supplied to the heater includes the heat flux through forced convection for heat exchange between the Si microchannel surface and the working fluid, as well as the heat flux through axial conduction inside the Si microchannel substrate. To avoid the axial conduction effect, the method whereby heat flux is supplied to the heater has not been used in the present study. The heat flux exchanged at the microchannel surface, *q* is obtained from the temperature difference at the fully developed flow region,


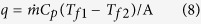


where *C*_*p*_ is the specific heat of the liquid and A is the equivalent surface area for heat transfer. The mean heat transfer coefficient and the Nusselt number are obtained as follows.


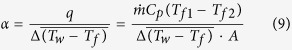



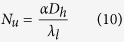


where 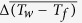
is the mean temperature difference between the channel wall and the working fluid.

**Figure 6:**
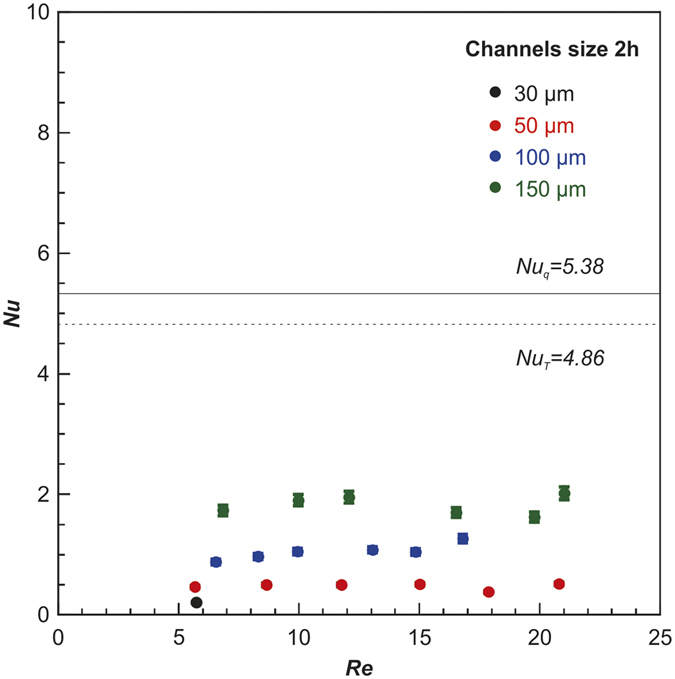
Experimental Nusselt numbers N_u_ vs. Reynolds number R_e_ in microchannels.

Figure 6 shows the experimentally obtained Nusselt numbers for the microchannels with hydraulic diameter *D*_*h*_ of 60 μm, 100 μm, 200 μm, and 300 μm, respectively. The experimental Nu numbers are much lower than the theoretical values of both Nu_q_ (constant heat flux) and Nu_T_ (constant surface temperature) based on the continuum boundary condition. The deviations between the experimental Nu and theoretical Nu increase as the hydraulic diameter of the microchannel decreases.

### Scale effects of interfacial resistances

The interfacial resistance (i.e., *l*_*s*_ and *l*_*k*_) can be estimated from the difference between continuum theory and the experimental results. Using Eq. (4) and the experimental mean P_o_ (=*fR*_*e*_), *l*_*s*_ can be estimated as follows:


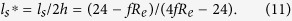


Similarly, *l*_*k*_ can be estimated using the experimental mean N_u_ and Eq. (5):


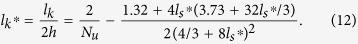


**Figure 7:**
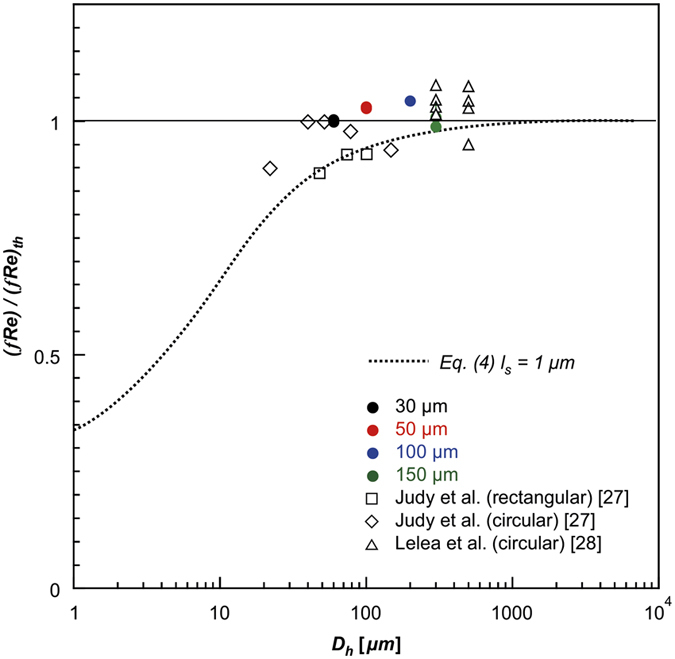
Scale effect of slip length on hydrodynamic resistance in microchannels.

**Figure 8:**
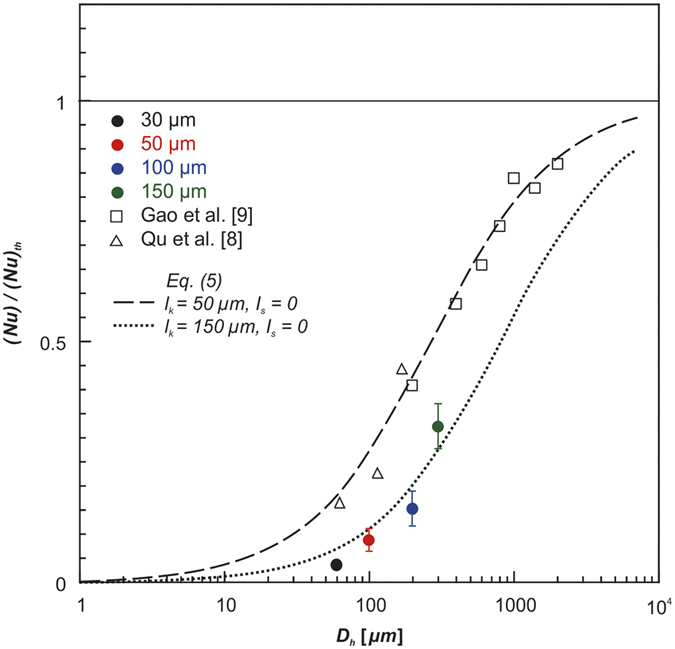
Scale effect of thermal slip length on convective heat transfer in microchannels.

Next, the forced convective heat transfer characteristics of the single-phase laminar flow in a parallel-plate microchannel are investigated experimentally. Figures 7 and 8 illustrate the experimental results and theoretical predictions, respectively, to clarify the scale effect of the hydraulic diameter on forced convection in microchannels. The theoretical P_o_ is *(fR*_*e*_)_*th*_ = 24, 64, and 57 for the parallel-plate channel, circular tube of refs 27 and 28, and the rectangular channel of Ref. 27, respectively (Fig. 3)^27^,^28^. The slip length of the water and silicon oxide interface in the present study can be assumed to be 0 because the experimental results agree well with the theoretical predictions. However, the experimental results obtained by Judy *et al*.^27^ in rectangular channels decrease with decreasing hydraulic diameter, which agrees fairly well with the theoretical prediction of *l*_*s*_ = 1 μm, for which the error is less than 2%.

In contrast to the foregoing results, the experimental N_u_ in Fig. 8 is significantly lower than the theoretical N_u_ under the no-slip boundary condition. The experimental N_u_ decreases with decreasing hydraulic diameter, whereas the discrepancy decreases with increasing hydraulic diameter, which is consistent with the trends reported in the literature^8^,^9^. The experimental N_u_ obtained in this study agrees well with the theoretical prediction of *l*_*s*_ = 0 μm and *l*_*k*_ = 150 μm, while those reported by Qu *et al*.^8^ and Gao *et al*.^9^ agree well with the theoretical prediction of *l*_*s*_ = 0 μm and *l*_*k*_ = 50 μm. In other words, the slip length and thermal slip length can no longer be ignored when these lengths are comparable with the hydraulic diameter. Therefore, we conclude that the scale effect explains the difference between the predictions of continuum theory and the experimental results.

Surface roughness^19^,^20^ exerts significant effects on forced convection heat transfer in microchannels. Moreover, surface wettability strongly affects convective heat transfer in microchannels^29^, and effective slip and friction reduction in nanograted superhydrophobic microchannels have been reported^30^. The effects of roughness and wettability, which are types of interfacial resistance, can be expressed using slip length and thermal slip length when the continuum boundary condition fails. Additional theoretical, molecular dynamics simulation^26^,^31^,^32^,^33^,^34^, and experimental studies^35^ on interfacial resistance are warranted to further clarify the mechanism.

## Summary

We studied the scale effect of the boundary condition at the solid–liquid interface on the single-phase convective heat transfer characteristics in microchannel or nanochannel flow. We have shown that the increasing inaccuracy of the predictions of classical theory with a decrease in the hydraulic diameter is due to the breakdown of the continuum solid–liquid boundary condition in microchannels. In other words, the solid–liquid interfacial resistance, which can be expressed as the slip length and thermal slip length, cannot be ignored when these lengths are comparable with the hydraulic diameter. Using the theoretical P_o_ and N_u_ derived under the slip boundary condition at the solid–liquid interface, we can estimate the slip length and thermal slip length at the solid–liquid interface.

## Additional Information

**How to cite this article**: Nagayama, G. *et al*. Scale effect of slip boundary condition at solid–liquid interface. *Sci. Rep.* **7**, 43125; doi: 10.1038/srep43125 (2017).

**Publisher's note:** Springer Nature remains neutral with regard to jurisdictional claims in published maps and institutional affiliations.

## Figures and Tables

**Table 1:** Nusselt and Poiseuille numbers for a fully developed laminar flow in various channels^6^.

Channels	N_uq_	N_uT_	N_uq_/N_uT_	P_o_
Parallel plates 1-side heated/1-side insulated 2-side heated	5.388.24	4.867.54	1.111.09	2424
Square	3.63	2.98	1.22	56
Circular	4.36	3.66	1.19	64

N_uq_ = Nusselt number at uniform surface temperature; N_uT_ = Nusselt number at uniform heat flux; P_o_ = Poiseuille number.
